# The Chief Diploma Vender Gone

**Published:** 1880-09

**Authors:** 


The Chief Diploma Vender Gone.—The "dean" and "professor" of the Philadelphia "diploma mill," is reported to have passed to his account, by his own act, and it is to be hoped that others engaged in similar outrages against humanity will cease the nefarious traffic, or follow the final example of their infamous confrere and predecessor? All such, and similar "deans" and "professors" now engaged in the traffic may rest assured that ere long their sins will find them out, and then it will be better for those they leave behind "had they never have been born, or that a mill stone were hanged about *their* necks" as their conscience-stricken bodies are plunged into the deep waters of the Delaware, or other stream, and their guilty souls into the Stygian river! Should there be one great sin more deep and damning than any other, it must be that of those who deliberately, and for money, license the ignorant and unprincipled who go forth to rob and slay their fellow men, under the authority of a "diploma" from their hands! This infamy will doubtless be held by the great Jehovah for special retribution. Such fiends will find "free thinking" and "Ingersolism", a sorry shield for their protection against the wrath of an offended Deity, ere they reach the Plutonian shore!

